# The Utilization of Dual Second Sacral Alar‐Iliac Screws for Spinopelvic Fixation in Patients with Severe Kyphoscoliosis

**DOI:** 10.1111/os.13348

**Published:** 2022-06-13

**Authors:** Ziyang Tang, Zongshan Hu, Zezhang Zhu, Jun Qiao, Saihu Mao, Chen Ling, Yong Qiu, Zhen Liu

**Affiliations:** ^1^ Department of Spine Surgery, Nanjing Drum Tower Hospital The Clinical College of Nanjing Medical University Nanjing 210008 China; ^2^ Department of Spine Surgery, Nanjing Drum Tower Hospital The Affiliated Hospital of Nanjing University Medical School Nanjing 210008 China

**Keywords:** Bone screw, Kyphoscoliosis, Pelvic fixation, Spinal fusion, Three‐dimensional CT reconstruction

## Abstract

**Objectives:**

As a new pelvic fixation technique, the dual S2AI screws fixation technique could provide highly stable distal strength, and have wide clinical prospect in the correction of severe kyphoscoliosis. However, the ideal trajectory parameters, indications and clinical outcomes of this technique have not been reported so far. This study aimed to determine the anatomical parameters of dual S2AI screws in the normal Chinese adult population, investigating the indications of this technique and evaluating the feasibility and clinical outcomes.

**Methods:**

Fifteen males and 15 females with normal pelvis underwent a pelvic CT scan to determine ideal dual S2AI screws trajectories. Sagittal angle (SA), transverse angle (TA), maximal length (ML), sacral length, and skin distance were measured. Subsequently, we retrospectively reviewed the data of 16 patients (seven males and nine females) who underwent dual S2AI screw fixation and 23 patients who underwent single S2AI screw fixation between January 2014 and December 2019. Preoperative, postoperative, and latest follow‐up measurements of Cobb angle, coronal balance (CB), spinal pelvic obliquity (SPO), and regional kyphosis (RK) were obtained. The mean follow‐up time was 16.7 ± 7.1 months (range: 12–30 months). Independent *t*‐test was used to determine the difference in the analysis of the trajectories. The paired sample non‐parametric Wilcoxon test was performed to assess the changes in radiographic parameters between different time points and different groups.

**Results:**

For both male and females, the proximal S2AI screws had significantly higher TA and ML, but a lower SA than distal screws. Females showed significantly more caudal (SA: 25.03° ± 2.32° *vs.* 29.82° ± 2.47°, *t* = 7.742, *P* < 0.001) trajectories of distal screw. Additionally, ML in the females were significantly shorter than that in males (106.81 mm ± 6.79 mm *vs.* 101.63 mm ± 6.55 mm, *t* = 3.007, *P* = 0.003, 124.41 mm ± 7.57 mm *vs.* 116.23 mm ± 7.03 mm, *t* = 4.337, *P* < 0.001). Eight had unilateral and eight had bilateral dual S2AI screw placement. Respectively, both the single S2AI and dual S2AI groups showed significant postoperative improvement of Cobb angle, RK angle and SPO angle. In patients with dual S2AI screws fixation, two patients found that screws loosening occurred in one of dual screws at 1‐year follow‐up, and in patients with single S2AI screws fixation, six patients found screw loosing as well as two patients found screw breakage at 1‐year follow‐up. None of all patients had any prominent loss of correction.

**Conclusion:**

The ideal trajectory of dual S2AI screw could be well established. The dual S2AI screw fixation technique is feasible in patients with severe kyphoscoliosis, and provides satisfactory correction of deformity with few postoperative complications.

## Graphical abstract

In this study, we verified the feasibility of the dual S2AI screws by constructing trajectories in the normal adult pelvis by 3D‐CT, and evaluated its clinical outcomes in 16 patients treated with this technique. Our study elucidated the ideal trajectory parameters of dual S2AI screws in normal pelvis, and confirm the favorable clinical outcomes of this technique in severe kyphoscoliosis. Our findings may help understand the technical essential, advantages and indications of this new technique.

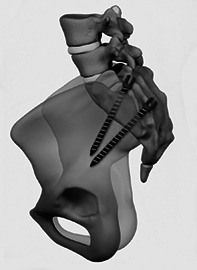

## Introduction

Establishing strong and solid fusions of lumbosacral spine is a challenging aspect of spine surgery.^1^, ^2^, ^3^, ^4^, ^5^ Several factors contribute to the high rates of instrumentation failure, including complex lumbosacral anatomy, poor bone quality, and presence of substantial biomechanical forces across the lumbosacral junction.^3^, ^4^ Studies have shown that fusion to L5 or S1 instead of the pelvis is associated with a high rate of pseudarthrosis in long‐segment fusions.^5^, ^6^ Therefore, spinopelvic fixation is regarded as an efficient technique that helps achieve strong fusions of the lumbosacral spine with low rate of complications.^7^ Sponseller *et al*.^8^ have shown that the second sacral alac‐iliac (S2AI) fixation technique, with the sacrum as the starting point, could provide substantial mechanical strength.

Since its introduction, the S2AI fixation technique has been widely utilized in the treatment of spinal deformity, lumbopelvic fracture subluxations, malignant tumor of pelvis, non‐unions, pathologic fractures and high‐grade spondylolisthesis.^9^ Compared to traditional spinopelvic fixation technique, S2AI screw fixation extends across the lumbosacral junction and provides higher fixation strength, along with fewer complications.^7^, ^9^ In our previous studies, we have shown that S2AI screw fixation technique provides favorable clinical outcomes in congenital scoliosis (CS), neuromuscular scoliosis (NMS) and degenerative scoliosis (DS).^7^, ^10^

Studies on the S2AI screw fixation technique have hitherto focused on evaluating the reliability of the single S2AI screw fixation technique, feasibility of placing S2AI screws with free‐hand technique or navigation system, and the long‐term clinical outcome in patients.^7^, ^11^ However, in clinical practice, spine surgeons often observe that single S2AI screw fixation may not provide efficient strength in some patients with severe kyphoscoliosis.^12^ Recently, Park *et al*.^5^ proposed a new type of S2AI screw fixation technique, involving the use of bilateral dual S2AI screws and found that this technique provided highly stable distal strength. However, since their study was a case report, they were not able to demonstrate whether the dual S2AI screw fixation technique would be effective in patients with severe kyphoscoliosis.

In this study, we aimed to determine the anatomical parameters in normal Chinese adult population by constructing simulative trajectories for the normal pelvis in 30 normal adults, using 3D reconstruction of CT data. Additionally, we aimed to identify the indications for dual S2AI screw technique and evaluate the feasibility and clinical outcomes of this technique by retrospectively reviewing the clinical data of patients with severe kyphoscoliosis who underwent dual S2AI screw fixation.

## Materials and Methods

### Patients Selection

The study protocol was approved by the institutional review board of our hospital, and the IRB/IEC number is 2021‐LCYJ‐DBZ‐05. Informed consent was obtained from all patients in this study for all procedures undertaken and all data obtained. Adults with normal pelvis who underwent pelvic CT scans between January 2014 and December 2019 were identified in our image processing system (Picture Archiving and Communication Systems, PACS). All patients with metabolic bone diseases, pelvic fractures, pelvic malignant tumor, congenital dislocation of the hip (CDH), or history of instrumentation surgeries were excluded from the study, and 30 adults were chosen for this study.

In addition, the medical records of 39 patients with severe kyphoscoliosis who underwent single or dual S2AI screw fixation between January 2014 and December 2019 were obtained from hospital database, and the data were retrospectively reviewed. The inclusion criteria were as follows: (i) fusion with pelvic fixation using single or dual S2AI screw fixation; (ii) availability of complete radiological data; and (iii) minimum of 1‐year follow‐up. Patients who met the following criteria were excluded: (i) diagnosis of pelvic fractures, malignant tumors or other diseases; (ii) history of undergoing a revision surgery for previous S2AI screw breakage; and (iii) non‐availability of complete clinical or radiological data for the follow‐up period. Meanwhile, on the basis of the different pelvic fixation, the patients were divided into two groups: single S2AI group and dual S2AI group.

Data on the following surgical parameters were collected in our study: levels fused, number of S2AI screws, utilization of iliac screw or iliosacral screw and surgical category (primary or revision). In addition, details on the utilization of other surgical techniques such as posterior column osteotomy (PCO), pedicle subtraction osteotomy (PSO), and transforaminal lumbar interbody fusion (TLIF) were also recorded.^8^

### Construction of Trajectories and Measurement

**Fig. 1:**
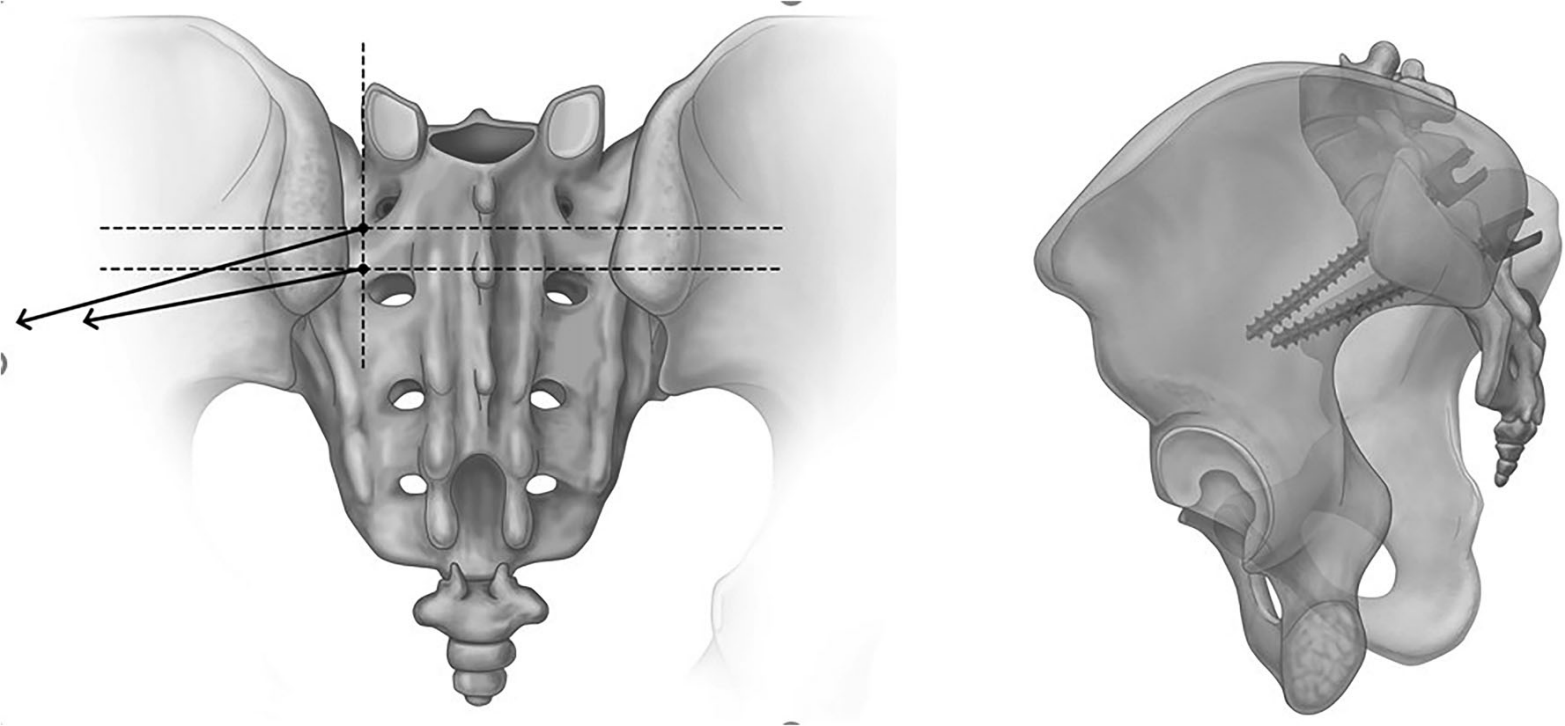
The anchoring point was defined as the point l mm below and 10 mm lateral to the S1 dorsal foramen for the proximal S2AI screw, and l mm above and 10 mm lateral to the S2 dorsal foramen for the distal S2AI screw.

All included patients had undergone pelvic CT scans by a 32‐slice CT device (Siemens company, Munich, Germany) with a layer thickness of 1.0 mm; voltage 150 kV; and current 220 mA. The 3D reconstruction was performed using Lightspeed workplace (General Electric Company, Boston, MA, USA). With the following setting: pitch of 1 mm and threshold of 300 HU, CT imaging planes were rotated until the greatest length and width of the osseous channel were obtained, in order to determine ideal S2AI trajectories. The anchoring points of the dual S2AI screws used in our study were different from those defined by Park *et al*.^5^ We selected the point that was l mm below and 10 mm lateral to the S1 dorsal foramen as the anchoring point of the proximal S2AI screw; and a point that was l mm above and 10 mm lateral to the S2 dorsal foramen as the anchoring point of the distal S2AI screw (Fig. 1).

**Fig. 2:**
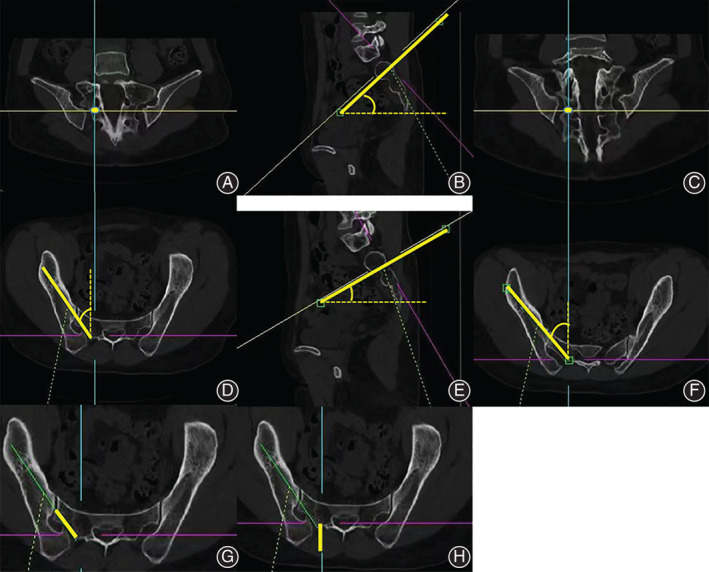
The schematic diagram of trajectories construction and measurement. (A, C) The anchoring points of the dual S2AI screws; (A, B, D) The virtual trajectory of the proximal S2AI screw; (C, E, F) The virtual trajectory of the distal S2AI screw; (B, E) Sagittal angle (SA), caudal trajectory angulation in the sagittal plane; (D, F) Transverse angle (TA), lateral trajectory angulation in the transverse plane; maximal length (ML), maximal distance of the trajectory from S2 ala to the anterior inferior iliac spine; (G) Sacral length (SL), intra‐sacral trajectory length; H: Skin distance (SD), the distance of the starting point lateral from the skin.

Subsequently, bilateral measurements were obtained for the following radiological parameters: (i) sagittal angle (SA), caudal trajectory angulation in the sagittal plane; (ii) transverse angle (TA), lateral trajectory angulation in the transverse plane; (iii) maximal length (ML), maximal distance of the trajectory from S2 ala to the anterior inferior iliac spine; (iv) sacral length (SL), intra‐sacral trajectory length; and (v) skin distance (SD), the distance of the starting point lateral from the skin (Fig. 2).

### Surgery Technique

The patients were placed in the prone positioned; after exposing the target levels, pedicle screws the S2AI screws were inserted into the fused segments using the free‐hand technique. The O‐arm Surgical Imaging System/StealthStation image navigation (Medtronic, Minneapolis, MN, USA), was used for the insertion of the single or dual S2AI screws. The StealthStation reference frame was fixed to the posterior superior iliac spine (PSIS), and then the CT scan was performed using O‐arm Surgical System. Subsequently, the StealthStation navigation system was used for 3D reconstruction of the images, and to provide guidance for the anchoring points and trajectories.^7^ Once the ideal trajectories were identified, single or dual S2AI screws with appropriate length were inserted into the patients' pelvis.

On the basis of previous studies and our experience, in patients with severe pelvic obliquity, especially in neuromuscular scoliosis (NMS), the accurate placement of S2AI screws remains a huge challenge.^13^, ^14^ Because of the severe obliquity and rotation of pelvis, the complex anatomical structures around, as well as the poor bone quality, the placement of dual S2AI screws could be infeasible sometimes, even under the guidance of O‐arm navigation system.^8^, ^14^, ^15^ In addition, this phenomenon usually occurred in the low side of pelvis, and then the dual S2AI screws would be only placed in the high side of pelvis for correction of pelvic obliquity.

Once all the screws were inserted at the planned levels, PCOs or PSO was performed for correction of kyphoscoliosis as scheduled. If necessary, TLIF would be performed at the L5/S1 level for establishing circumferential fusion, anterior column support, and a horizontal L5. Pre‐bent titanium rods were installed after placement of all other components. Further, distraction on the concave side and compression on the convex side were performed to correct the spinal deformity.

### Radiographic and Clinical Measurements

To access the radiographic outcomes, all patients underwent preoperative, postoperative, and latest follow‐up full‐spine anteroposterior radiograph while standing or sitting. All parameters were measured by the same spinal surgeon using Surgimap Software (Nemaris, New York, USA). The following radiographic parameters were measured: (i) Cobb angle, major coronal curves were measured with posteroanterior standing radiographs; (ii) spinal pelvic obliquity (SPO) angle, the angle between the line perpendicular to the iliac crest line and the line between the spinous process of T1 and the middle of the sacroiliac line^13^, ^16^; (iii) regional kyphosis (RK): the angle formed between the superior endplate of the upper end vertebra and the inferior endplate of the lower end vertebra; and (iv) coronal balance (CB), the perpendicular distance between the C7 plumbline and the central sacral vertical line (C7PL–CSVL).

Shillingford *et al*.^11^ introduced a new concept as screw breach in order to evaluate the accuracy of S2AI screw placement. Breaches was defined as perforation of cortical bone resulting in any portion of the screw lying outside the confines of the pelvis, and were graded on a scale of 0 to 3, with grade 0 defined as no breach and grades 1 to 3 defined according to the breach distance: grade 1 (mild), a breach distance of <3 mm; grade 2 (moderate), 3 to 6 mm; and grade 3 (severe), >6 mm. According to post‐operative CT images, the accuracy of S2AI screws placement could be graded by Breach Grading.^11^

Furthermore, the Scoliosis Research Society (SRS)‐22 questionnaire was employed for the evaluation of the patient‐reported outcomes. Data on perioperative and postoperative complications were also recorded.

### Statistical Analysis

Statistical analysis was performed using the SPSS statistical package (version 26.0, IBM, Armonk, NY, USA). Continuous variables are represented as a mean ± standard deviations. To account for the effect of the differences in morphology between the male and female pelvis on the analysis of the trajectories, independent *t*‐test was used to detect possible divergence in the above‐mentioned data for differences related to sex of the subject or screw types. For the analysis of the radiographic and clinical measurements, repeated measures of ANOVA test was used to determine the overall differences between the preoperative and postoperative, and latest follow‐up measurement. The paired sample non‐parametric Wilcoxon test was performed to assess the changes in radiographic parameters between the pre‐, post‐operation and the last follow‐up. Fisher exact test was used to determine the risk factors for complications. A *P* value of <0.05 was considered to indicate statistical significance.

## Result

### The Anatomical Parameters of Dual S2AI Screws in Adults with Normal Pelvis

Thirty adults (15 males and 15 females) whose pelvis were confirmed to be normal and symmetric by CT scans were identified and their data was analyzed. The mean age of the enrolled subjects was 35.7 ± 14.7 years (range: 20–65 years). Bilateral construction of virtual trajectories of dual S2AI screws using pelvic CT scans was possible for all patients. The anatomical parameters were measured and analyzed by 3D radiographic construction (Table 1). Compared to the distal S2AI screws, the proximal S2AI screws had significantly lower TA (36.91° ± 2.42° *vs*. 41.78° ± 2.57°, *t* = 7.712, *P* < 0.001; 36.73° ± 2.07° *vs*. 41.15° ± 2.73°, *t* = 7.073, *P* < 0.001) and ML (106.81 mm ± 6.79 mm *vs*. 124.41 mm ± 7.57 mm, *t* = 9.480, *p* < 0.001; 101.63 mm ± 6.55 mm *vs*. 116.23 mm ± 7.03 mm, *t* = 8.328, *p* < 0.001), but higher SA (43.35° ± 4.11° *vs*. 25.03° ± 2.32°, *t* = 21.261, *P* < 0.001; 42.88° ± 3.71° *vs*. 29.82° ± 2.47°, *t* = 16.054, *P* < 0.001) and SL (28.87 mm ± 3.79 mm *vs*. 26.33 mm ± 3.16 mm, *t* = 2.819, *P* = 0.007; 30.74 mm ± 4.25 mm *vs*. 27.76 mm ± 3.03 mm, *t* = 3.127, *P* = 0.003). In addition, the ML of the dual screws were significantly shorter in females than in males (ML: 106.81 mm ± 6.79 mm *vs*. 101.63 mm ± 6.55 mm, *t* = 3.007, *p* = 0.003, 124.41 mm ± 7.57 mm *vs*. 116.23 mm ± 7.03 mm, *t* = 4.337, *P* < 0.001). In particular, the distal S2AI screw was significantly more caudal (higher SA) in females than in males (SA: 25.03° ± 2.32° *vs*. 29.82° ± 2.47°, *t* = 7.742, *P* < 0.001).

**TABLE 1:** The parameters of dual S2AI screws trajectories

Parameters	Proximal S2AI screw	Distal S2AI screw	*t* value	*p* value
	Male (*n* = 15 × 2)			
SA (°)	43.35 ± 4.11	25.03 ± 2.32	21.261	0.001
TA (°)	36.91 ± 2.42	41.78 ± 2.57	7.712	0.001
ML (mm)	106.81 ± 6.79	124.41 ± 7.57	9.480	0.001
SL (mm)	28.87 ± 3.79	26.33 ± 3.16	2.819	0.007
SD (mm)	49.73 ± 12.54	46.53 ± 9.37	1.120	0.267
	Female (*n* = 15 × 2)			
SA (°)	42.88 ± 3.71	29.82 ± 2.47 *	16.054	0.001
TA (°)	36.73 ± 2.07	41.15 ± 2.73	7.073	0.001
ML (mm)	101.63 ± 6.55*	116.23 ± 7.03*	8.328	0.001
SL (mm)	30.74 ± 4.25	27.76 ± 3.03	3.127	0.003
SD (mm)	50.68 ± 9.93	47.09 ± 11.44	1.298	0.199

“*”: which compare with male's parameter *P* < 0.05.

S2AI: second sacral alar‐iliac; SA: sagittal angle; TA: transverse angle; ML: maximal length; SL: sacral length; SD: skin distance.

### Demographics Data of Patients Who Underwent Dual S2AI Screw Fixation

The demographic and clinical data of 39 patients, including 14 males and 25 females, who underwent single or dual S2AI screw fixation between January 2014 and December 2019 were retrospectively investigated in this study. The mean age of the patients was 36.5 ± 20.3 years old (range: 12–60 years). The mean follow‐up time was 16.7 ± 7.1 months (range: 12–30 months). The pathogenesis was osteopsathyrosis and viral encephalitis in 1 case; neurofibromatosis type‐1 (NF‐1), Chiari malformation, and transverse myelitis in two cases each; spinal muscular atrophy (SMA) in three cases, iatrogenic kyphosis in three cases; cerebral palsy in four cases; degenerative scoliosis (DS) in eight cases and poliomyelitis in 13 cases. Furthermore, 14 patients had incomplete paraplegia, seven had paraplegia of bilateral lower limbs, two had multiple paravertebral neurofibromas, and 12 patients had severe lower back pain. The demographics data of patients in two groups were recorded in Table 2, and the specific data of patients in the dual S2AI group were recorded in Table 3.

**TABLE 2:** The demographic data and baseline parameters of all patients

	Single S2AI group	Dual S2AI group
Total	23	16
Male	7	7
Female	16	9
Age	39.7 ± 18.1	30.3 ± 16.2
Etiology		
Neurofibromatosis type‐1	1	1
Chiari malformation	1	1
Osteopsathyrosis	0	1
Viral encephalitis	0	1
Cerebral palsy	3	1
Transverse myelitis	1	1
Spinal muscular atrophy (SMA)	1	2
Iatrogenic kyphosis	0	3
Degenerative scoliosis (DS)	8	0
Poliomyelitis	8	5
Operation time (h)	4.3 ± 1.2 *	5.1 ± 0.7
Blood loss volume (mL)	1372 ± 535	1525 ± 485
Fused levels	12.8 ± 2.4	13.3 ± 2.0
3‐Coloum osteotomies	7	3
TLIF	5	3

* Statistically significant between single S2AI group and dual S2AI group in operation time, *t* = 2.393, *P* = 0.02.

**TABLE 3:** Diagnosis and surgical data of all the patients in dual S2AI group

No.	Gender	Age	Diagnosis	Revision	Fusion segments	Number of S2AI screws	Number of Rod	Other technique	TLIF	Follow‐up time
1	M	20	NF‐1	No	16 T4‐S2	2	3	No	No	12 months
2	M	20	Chiari malformation, myelomeningocele	No	13 T7‐S2	2	3	1 ISS screw	No	24 months
3	F	18	Poliomyelitis	Yes	15 T5‐S2	3	5	1 IS screw	No	12 months
4	M	39	Poliomyelitis	No	10 T10‐S2	4	4	No	No	20 months
5	F	18	DMD	No	16 T4‐S2	3	4	No	No	12 months
6	F	18	Osteopsathyrosis	No	11 T9‐S2	4	4	No	No	12 months
7	F	54	Poliomyelitis	No	12 T8‐S2	4	4	No	Yes	12 months
8	M	16	Viral encephalitis	No	15 T5‐S2	3	4	No	No	12 months
9	F	12	Paraplegia after acute myelitis	No	15 T5‐S2	3	4	No	No	12 months
10	M	42	Iatrogenic kyphosis	Yes	12 T8‐S2	3	4	1 IS screw	Yes	16 months
11	F	60	Iatrogenic kyphosis	Yes	11 T9‐S2	4	4	No	Yes	30 months
12	F	54	Poliomyelitis	No	12 T8‐S2	4	4	No	No	15 months
13	F	43	Cerebral palsy	No	15 T5‐S2	4	4	No	No	18 months
14	M	19	Spinal muscular atrophy (SMA)	No	14 T6‐S2	3	4	No	No	12 months
15	F	16	Spinal muscular atrophy (SMA)	No	15 T5‐S2	4	4	No	No	12 months
16	M	36	Iatrogenic kyphosis	Yes	11 T9‐S2	4	4	No	No	15 months

S2AI: second sacral alar‐iliac; DMD: Duchenne muscular dystrophy; NF‐1: Neurofibromatosis type 1.

### Surgical Data

The demographic and clinical data of the patients in two groups were assessed (Tables 2 and 3). In the dual S2AI group, the mean operation time was 5.1 ± 0.7 h (range: 4.5–6.8 h), which was significantly longer than that in single S2AI group (5.1 ± 0.7 *vs*. 4.3 ± 1.2, *t* = 2.393, *P =* 0.02). However, there was no significantly difference in blood loss volume and fused levels between two groups (1525 ± 485 *vs*. 1372 ± 535, *t* = 0.912, *P =* 0.368; 13.2 ± 2.0 *vs*. 12.8 ± 2.4, *t* = 0.684, *P =* 0.498). In the dual S2AI group, a total of 54 S2AI screws that were used in the patients, unilateral dual S2AI screws were placed in eight patients, and bilateral dual S2AI screws in eight patients (Table 3).

The screw breach score system was applied to evaluate the accuracy of S2AI screws. In the dual S2AI group, the trajectories of the S2AI screws were graded 0 for 48 of the 54 screws (88.89%) and graded 1 for the remaining six screws (11.11%). Additionally, in the single S2AI group, the trajectories of the S2AI screws were graded 0 for 37 of the 46 screws (80.43%) and graded 1 for the remaining nine screws (19.57%). All trajectories were confirmed to be accurate and safe.^11^

### Radiographic Outcomes

**Fig. 3:**
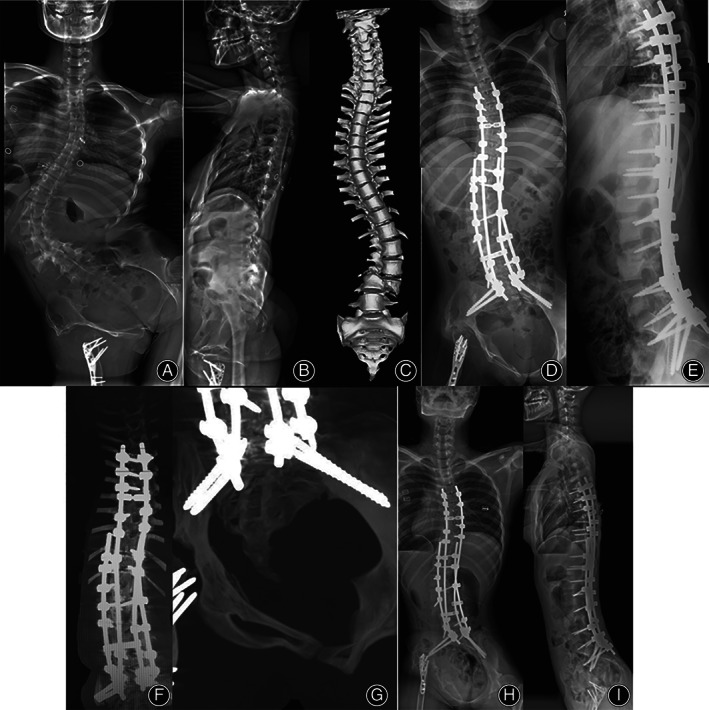
Female, 18 years old, diagnosed with osteopsathyrosis. (A, B) The pre‐operative anteroposterior radiograph showed that Cobb angle was 87.2°, SPO was 51.3° and CB was 42.1 mm. (C) pre‐operative CT scans showed multiple fractures of lower limbs and L3/4 fracture dislocation. (D, E) Postoperative anteroposterior radiograph showed significant correction of kyphoscoliosis and PO, the Cobb angle was 25.4°, SPO was 18.7° and CB was 16.2 mm. (F, G) Post‐operative CT scans showed the placement of bilateral dual S2AI screws. (H, I) At 1‐year follow‐up, the anteroposterior radiograph showed the correction maintained well, the Cobb angle was 27.2°, SPO was 16.1° and CB was 10.3 mm.

**Fig. 4:**
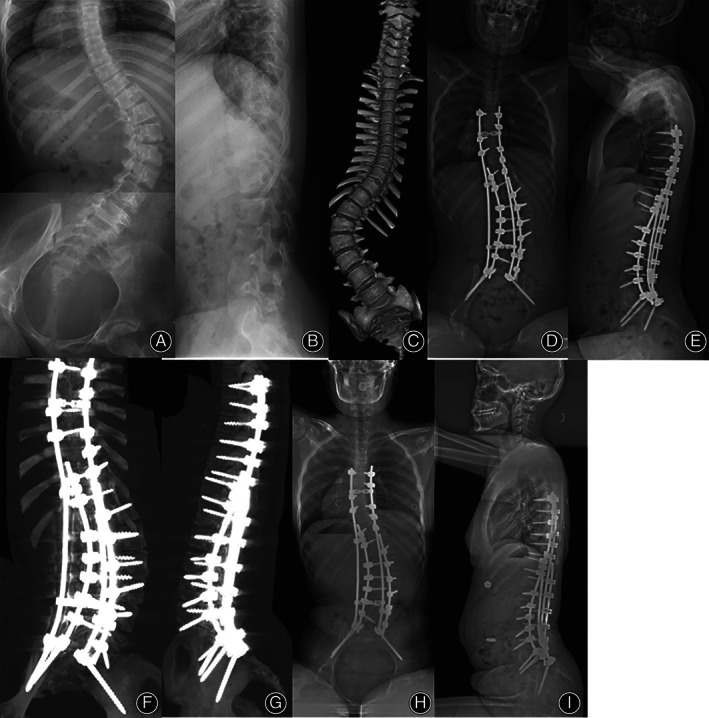
Female, 12 years old, diagnosed with osteopsathyrosis. (A, B, C) The pre‐operative anteroposterior radiograph and CT scans showed that Cobb angle was 90.4°, SPO was 30.7° and CB was 36.4 mm. (D, E) Postoperative anteroposterior radiograph showed significant correction of kyphoscoliosis and PO, the Cobb angle was 34.2°, SPO was 12.1° and CB was 9.3 mm. (F, G) Post‐operative CT scans showed the placement of unilateral dual S2AI screws. (H, I) 1‐year follow‐up, the anteroposterior radiograph showed the Cobb angle was 35.1°, SPO was 14.3° and CB was 6.2 mm, the correction maintained well.

Measurements of the radiographic parameters obtained for all patients are presented in Table 4. There was no significant difference in the pre‐operative radiographic parameters between two groups. In both two groups, the Cobb angles for the major curve were improved significantly postoperatively (73.8° ± 34.9° *vs*. 34.2° ± 25.1°, *Z* = −2.965, *P* = 0.003 in the dual S2AI group; 65.4° ± 30.3° *vs*. 30.2° ± 27.9°, *Z* = −3.072, *P* = 0.002 in the single S2AI group), and the correction was maintained well at the last follow‐up (*P* > 0.05). In addition, there was a significant improvement for SPO angle, RK, and CB after surgery in both two groups (*P* < 0.01); moreover, there was no significant loss of correction at the last follow‐up (*P* > 0.05) (Figs Fig. 3 and Fig. 4). Between patients in the single S2AI group and the dual S2AI group, the main Cobb angle, SPO angle, CB and RK showed no significant difference postoperatively and at final follow‐up (*P* > 0.05).

**TABLE 4:** Comparison of radiographic outcomes between pre‐operation, post‐operation and last follow‐up

	Single S2AI group	Dual S2AI group	*Z* value	*P* value
Cobb angle (°)				
Pre‐op	65.4 ± 30.2	73.8 ± 34.9	−0.798	0.428
Post‐op	30.3 ± 27.9 *	34.2 ± 25.1 *	−0.445	0.657
Last follow‐up	32.2 ± 29.8 **	35.4 ± 26.4 **	−0.342	0.732
CB (mm)				
Pre‐op	37.2 ± 25.3	31.2 ± 31.0	0.655	0.511
Post‐op	18.7 ± 18.3 *	13.5 ± 17.3 *	0.883	0.378
Last follow‐up	23.7 ± 19.9 **	12.8 ± 17.1 **	1.671	0.095
SPO angle (°)				
Pre‐op	26.4 ± 20.3	30.9 ± 18.7	−0.693	0.487
Post‐op	16.2 ± 13.2 *	13.6 ± 9.0 *	−0.674	0.498
Last follow‐up	15.7 ± 10.9 **	14.0 ± 8.7 **	−0.516	0.607
RK (°)				
Pre‐op	45.4 ± 29.6	40.9 ± 34.2	0.434	0.664
Post‐op	20.7 ± 16.4 *	14.3 ± 16.3 *	−1.226	0.223
Last follow‐up	22.1 ± 15.2 **	15.1 ± 17.1 **	−1.314	0.187

S2AI: second sacral alar‐iliac; SPO: spinal pelvic obliquity; RK: regional kyphosis; CB: coronal balance.

* Statistically significant between pre‐operation and post‐operation, *P* < 0.05.

^**^ Statistically significant between post‐operation and last follow‐up, *P* < 0.05.

### Patient‐reported Outcomes

The patient‐reported outcomes were evaluated in the form of preoperative and postoperative health‐related quality of life (HRQoL) scores, as shown in Table 5. In both these two groups, the scores revealed a significant improvement of HRQoL scores of function, pain and self‐image (*P* < 0.05), while the mental health scores showed no significant difference (*t* = 0.959, *P* = 0.343 in single S2AI group; *t* = 1.874, *P* = 0.071 in dual S2AI group). Additionally, there was no significant difference in pre‐ and post‐operative HRQoL scores between these two groups (*P* > 0.05).

**TABLE 5:** Comparison of preoperative and postoperative SRS‐22 scores

	Single S2AI group	Dual S2AI group	*t* value	*p* value
Function				
Pre‐operation	2.6 ± 0.5	2.7 ± 0.6	0.547	0.588
Post‐operation	3.4 ± 0.6 *	3.5 ± 0.4 *	0.582	0.536
Self‐image				
Pre‐operation	3.0 ± 0.6	2.8 ± 0.4	1.163	0.252
Post‐operation	3.7 ± 0.4 *	3.9 ± 0.5 *	1.386	0.174
Pain				
Pre‐operation	2.8 ± 0.8	3.0 ± 0.7	0.807	0.414
Post‐operation	3.8 ± 0.6 *	4.1 ± 0.4 *	1.745	0.089
Mental health				
Pre‐operation	3.4 ± 0.6	3.3 ± 0.5	0.547	0.575
Post‐operation	3.6 ± 0.8	3.6 ± 0.4	0.000	1.000
Satisfaction				
	3.9 ± 0.7	4.0 ± 0.5	0.490	0.605

SRS: scoliosis research society.

* Statistically significant between pre‐operation and post‐operation, *P* < 0.05.

### Complications

In dual S2AI group, one of the patients developed infection, which was treated with multiple debridement and subsided without the need for the removal of the instrumentation. There was no case of neurovascular injury during the perioperative period. Additionally, two of the patients found that screws loosing occurred in one of dual screws at 1‐year follow‐up, but they had no symptoms. In addition, in single S2AI group, six patients found screw loosing as well as two patients found screw breakage at 1‐year follow‐up, and two patients underwent revision surgery finally. The incidence of implant‐related complications was higher in single S2AI group (8/23, 34.7%; 2/16, 12.5%), but there is no significant difference between two groups (*χ* ^*2*^ = 2.457, *p* = 0.117). Moreover, none of the patients developed instrumentation related complications including rod breakage, screw fracture and L5‐S1 pseudarthrosis.

## Discussion

In this study, we determined the feasibility of the dual S2AI screws fixation technique by constructing trajectories in the normal adult pelvis by 3D‐CT, and determine the indications of this technique, as well as evaluated its clinical outcomes in 16 patients treated with this technique. The results showed that this novel technique could provide a significant correction of kyphoscoliosis with less complications. During the follow‐up, the coronal and sagittal balance were well‐maintained. To our knowledge, after the case report by Park *et al*.,^5^ ours is the only study on this technique with a greater number of cases.

### The Anatomical Parameters of Dual S2AI Screws in Normal Chinese Adult Population

The dual S2AI screw fixation technique was first introduced by Park *et al*.^5^ in a case report. This technique facilitates the construction of a stable structure with high implant density in the lumbosacral region. On the basis of findings of previous studies,^7^, ^9^, ^14^ we chose the two anchoring points between the S1 and S2 dorsal foramen, and constructed the simulative trajectories from these points. Consistent with the technique elucidated by Park *et al*.,^5^ we maintained the anchoring points of dual S2AI screws in‐line with that of the S1 pedicle screws. As shown in Table 1, independent trajectories of the dual S2AI screws could be constructed without intersection. The trajectories of the proximal S2AI screw were more caudal and more inward than the distal screw, which a finding that has not been reported hitherto. This may be determined by the pelvic autologous anatomical morphology. Zhu *et al*.^15^ have reported that the trajectories of S2AI screw were more caudal in females; this finding was consistent with ours, with the trajectories of the distal S2AI screw being more caudal in females with a higher SA (25.03° ± 2.32° *vs*. 29.82° ± 2.47°, *t* = 7.742, *P* < 0.001).^15^ Furthermore, we also noticed that the difference of SA in the proximal S2AI screw was not statistically significant (43.35° ± 4.11° *vs*. 42.88° ± 3.71°, *t* = 0.465, *P* = 0.643).

Further, the differences in TA for males and females were also consistent with those reported by Zhu *et al*.^15^ Although there was some difference in TA, it was not statistically significant (36.91° ± 2.42° *vs*. 36.73° ± 2.07°, *t* = 0.310, *P* = 0.758; 41.78° ± 2.57° *vs*. 41.15° ± 2.73°, *t* = 0.920, *P* = 0.361). In addition, the distal S2AI screw had a lower SA (*t* = 21.261, *t* = 16.054; both *P* < 0.001) and a higher TA (*t* = 7.712, *t* = 7.703; both *P* < 0.001). The anatomical parameters of dual S2AI screws appeared to be complex, but these parameters tended to be coincident and steady. Therefore, accurate placement of the dual S2AI screws with free‐hand technique appeared to be feasible in normal adults.

### The Indications of the Dual S2AI Screws Fixation Technique in Severe Kyphoscoliosis

Several studies have demonstrated the advantages of the single S2AI screw fixation technique.^7^ Biomechanical studies have shown that S2AI screws could provide optimal lumbosacral trajectories between the iliac cortices down to the superior acetabular bone.^17^ Therefore, in the majority of patients, single S2AI screw fixation could provide distal fixation of adequate strength.^7^, ^14^ However, a recent 26‐month follow‐up study by Hyun *et al*.^12^ revealed that screw fracture and dislodgement of the set screw occurred in 7.9% (25/312) single S2AI screws, while distal device breakage occurred in 3.2% (5/156) patients. These findings suggested that the single S2AI screw fixation technique could still be associated with some mechanical defects, especially around the area where the screw head and rod were in contact and the durability of the set screw.

Low implant density could be associated with poor radiographic outcomes and a high rate of instrumentation related complications.^18^ Li *et al*.^18^ recommended the utilization of pelvic fixation technique in cases where the lumbar vertebrae could not accommodate pedicle screws for a solid and stable base distally. In addition, Yu *et al*.^19^ recommended that enhancement of lumbosacral implant density instead of the fixation strength of the single screws would be more effective in increasing the stability of lumbo‐iliac fixation. Accordingly, surgeons prefer the use of the dual iliac screw (DIS) fixation technique to achieve a strong spinopelvic fixation.^19^ Considering the biomechanical advantages of S2AI screw, we recommended the dual S2AI screws fixation technique over the DIS fixation technique for more stable fixation. Among our patients, one had NF‐1 and one had Chiari malformation. In these patients, multiple thoracolumbar vertebrae could not accommodate pedicle screws, and the pelvis could not accommodate a S2AI screw or iliac screw in the concave side due to the osseous destruction and morphological variation of vertebrae. As a remedial strategy to provide additional distal stabilization in these cases, dual S2AI screws were placed in the convex side to correct the spinopelvic deformity and achieve sufficient implant density.

The lumbosacral junction could be especially difficult to stabilize during fusion due to the high biomechanical loads and shear forces acting at this junction.^7^, ^14^ Therefore, many surgeons prefer multi‐rod construction in the lumbosacral region to achieve adequate biomechanical stability.^17^ Robert *et al*.^20^ noted that lumbosacral rod fracture (RF) occurred in 40% (6/15) patients in 2‐rod group but none in multi‐rod group. Similarly, Yamato *et al*.^21^ reported the incidence of RF was obviously lower in the 3‐rod group than in the 2‐rod group (68.0% *vs*. 39.1%). However, Shen *et al*.^22^ have attested to the difficulty in setting two ipsilateral rods each into different screw heads. This challenge could be overcome by the use of the dual S2AI screws fixation; this would allow the two S2AI screws on the same side to be connected to the same rod for stronger fixation, as well as to two different rods for further dispersal of the stress without complex connectors and extending multi‐rod construction to lumbosacral region. Meanwhile, the multi‐rod construction extending to the pelvis could provide a convenient solution to perform sequential correction in lumbosacral region. In several of our cases, the dual S2AI screws could be used to allow the construction of a multi‐rod structure across the lumbosacral region for more powerful fixation. The post‐operative radiographic parameters showed that good correction of the PO was achieved and coronal alignment was effectively reconstructed.

The “Kickstand rod” technique and “Tie rod” technique were previously used for the revision of DS.^23^ These techniques were more effective in providing the appropriate torque through an independent iliac screw and an accessory rod. Actually, the dual S2AI screws fixation technique had similar structural characteristics to the above‐mentioned techniques, and the S2AI screw had better mechanical strength than IS.^14^ Additionally, solid distal anchoring could allow surgeons to perform revision surgery without completely removing the initial implants.^7^, ^9^, ^14^ Three of our cases (cases 10, 11 and 16) underwent revision surgeries due to instrumentation failure. In both these patients, the previous surgeries had caused severe instability at the lumbosacral region. However, the dual S2AI screws fixation technique was successfully used in these revision surgeries to obtain stable fixation, correction of deformity and further reduction of the surgical wound.

On the basis of this study, in patients with severe kyphoscoliosis, we recommended that dual S2AI screws could be considered as a good option when a single S2AI screw could not provide enough mechanical strength, especially in these indications as follow: (i) necessity to ensure sufficient implant density; (ii) rigid lumbosacral deformity with severe coronal imbalance; and (iii) complex revision surgery.

### The Clinical Outcomes of the Dual S2AI Screws Fixation Technique

In our study, we utilized the dual S2AI screws fixation technique in 16 patients with severe kyphoscoliosis, which could provide significant correction of the Cobb angle, SPO angle, RK angle and CB. Additionally, the correction was successfully maintained well at the last follow‐up assessment. There was no significant difference between the two groups, which may be due to heterogeneous etiologies. Thereby, the dual S2AI screw fixation technique could afford satisfactory clinical outcomes in patients with severe kyphoscoliosis.^3^, ^8^, ^14^ Additionally, the trajectories of the dual S2AI screws were graded 0 for 48 of the 54 screws (88.89%) and graded 1 for the remaining six screws (11.11%), thus indicating that all trajectories were accurate.^11^ These results suggest that the dual S2AI screws fixation technique is accurate in patients with severe kyphoscoliosis.

Studies have shown that while the use of the single S2AI screw technique causes wound complications in 6.1–10% patients, 3.2–7.8% develop instrumentation‐related complications such as screw loosing, rod breakage, implant prominence, and so on.^12^, ^14^ In our study, deep wound infection occurred only in one patient and the wound healed well after debridement. In addition, two of the patients found that screw loosening occurred in one of the dual screws at 1‐year follow‐up. Notably, the remaining dual S2AI screw could provide adequate strength and maintain stabilization of distal construction. Apart from these, there were no other instances of instrumentation‐related complications. In our study, compared with the single S2AI group, the incidence of implant‐related complications was lower in dual S2AI group (8/23, 34.7%; 2/16, 12.5%), but there is no significant difference between two groups. In addition, as a common complication in sacropelvic fixation, Eiki *et al*.^24^ reported 4.2% patients with S2AI screw fixation could suffer from sacroiliac joint pain (SIJP); however, none of patients in our study developed SIJP during the follow‐up period. We believed that the second screw may act as an anti‐rotation screw, preventing the rudimentary movement of the sacroiliac join, and thereby decreasing the associated SIJP.^24^ Actually, in our study, the patients in the dual S2AI group had a lower post‐operative pain score than those in the single S2AI group (3.8 ± 0.6 *vs*. 4.1 ± 0.4, *t* = 1.745, *P* = 0.089), although there was no significant difference between two groups. Therefore, the utilization of dual S2AI screws is associated with a lower incidence of complications as compared to that observed with the use of a single S2AI screw.

### Limitations

This study on the dual screw fixation technique is the largest single‐center study of its kind. However, it does have some limitations. Some of the limitations are single‐center, retrospective design, and a small sample size. Therefore, more effective statistical analysis was not possible; more large‐scale and prospective studies are required for a deeper insight. In addition, the patients in this study were followed up for only a short term, and the incidence of some complications could not be accurately ascertained. Long‐term studies are necessary to clearly determine the clinical outcomes of dual S2AI fixation and the incidence of complications over time. Finally, in previous studies, Mazur *et al*.^25^ reported that patients with S2AI screw fixation could achieve an osseous union at L5‐S1 after a mean follow‐up of 24.8 months. However, in our study, the majority of patients only had a short‐term follow‐up less than 1.5 years, which was too early to evaluate the achievement of solid fusion on L5‐S1. Additionally, in our patients, the lumbosacral CT scans were not performed routinely during the short‐term follow‐up, unless the implant‐related complications occurred. Therefore, a longitudinal long‐term study including lumbosacral CT scans should be listed as important parts of the future studies to observe the clinical outcomes and to evaluate the fusion of lumbosacral junction.

### Conclusion

To conclude, our investigations showed that the ideal trajectory of dual S2AI screw fixation could be well established in the normal pelvis. Furthermore, our findings also indicate that dual S2AI screw fixation is a feasible technique in patients with severe kyphoscoliosis, providing satisfactory correction of coronal balance and sagittal alignment with few postoperative complications.

## Funding

This work was supported by National Natural Science Foundation of China (82072518).
